# Supplementary material for: Spatiotemporal reprogramming of differentiated cells underlies regeneration and neoplasia in the intestinal epithelium
Source: Nat Commun. 2022 Mar 21;13:1500. doi: 10.1038/s41467-022-29165-z (PMC8938507; doi:10.1038/s41467-022-29165-z)
Supplement: Supplementary file 1 — Supplementary Information [file 41467_2022_29165_MOESM1_ESM.pdf]

Supplementary Information

Spatiotemporal reprogramming of differentiated cells underlies regeneration and neoplasia in the intestinal epithelium

Tsunaki Higa, Yasutaka Okita, Akinobu Matsumoto, Shogo Nakayama, Takeru Oka, Osamu Sugahara, Daisuke Koga, Shoichiro Takeishi, Hirokazu Nakatsumi, Naoki Hosen, Sylvie Robine, Makoto M. Taketo, Toshiro Sato & Keiichi I. Nakayama

Supplementary Figures 1–8

Supplementary Table Information

Supplementary Figures

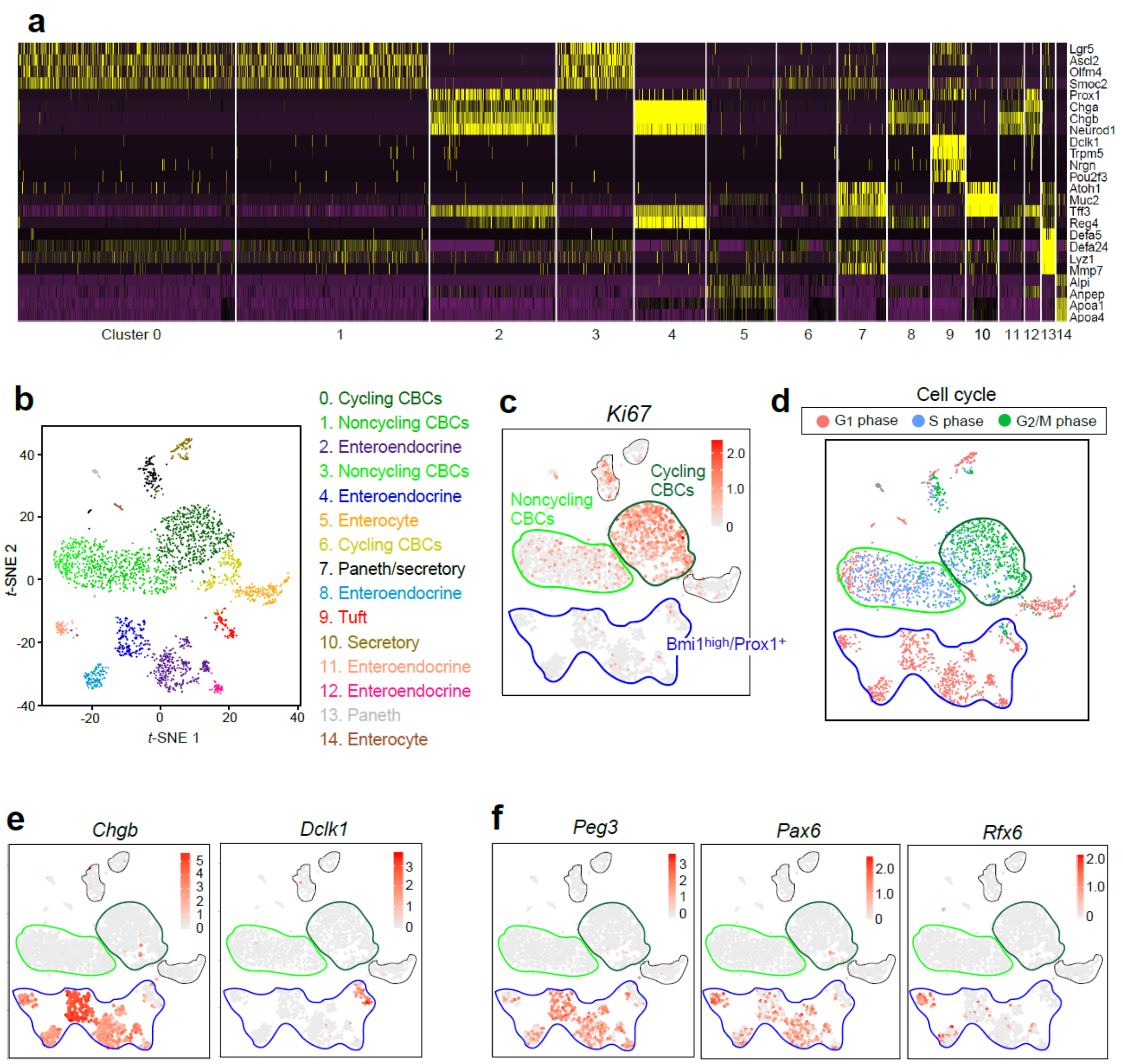

Supplementary Fig. 1 Expression of representative intestinal cell type markers in single-cell clusters. a Heat map showing the z-score for the expression level of known cell type–specific marker genes in crypt cell clusters from an scRNA-seq data set[14]. **b** Color-coded crypt cell clusters are shown on the *t*-SNE projection, with the identity of each cluster being based on the expression of known cell type–specific genes as shown in **a**. **c** Heat map showing the abundance of Ki67 mRNA overlaid on the *t*-SNE projection. **d** Results of cell cycle status scoring analysis of the scRNA-seq data by Seurat shown on the *t*-SNE projection. **e**, **f** Heat maps for the abundance of mRNAs for the representative EE cell marker Chgb or tuft cell marker Dclk1 (**e**) or for intestinal LRC markers (**f**) shown on the *t*-SNE projection.

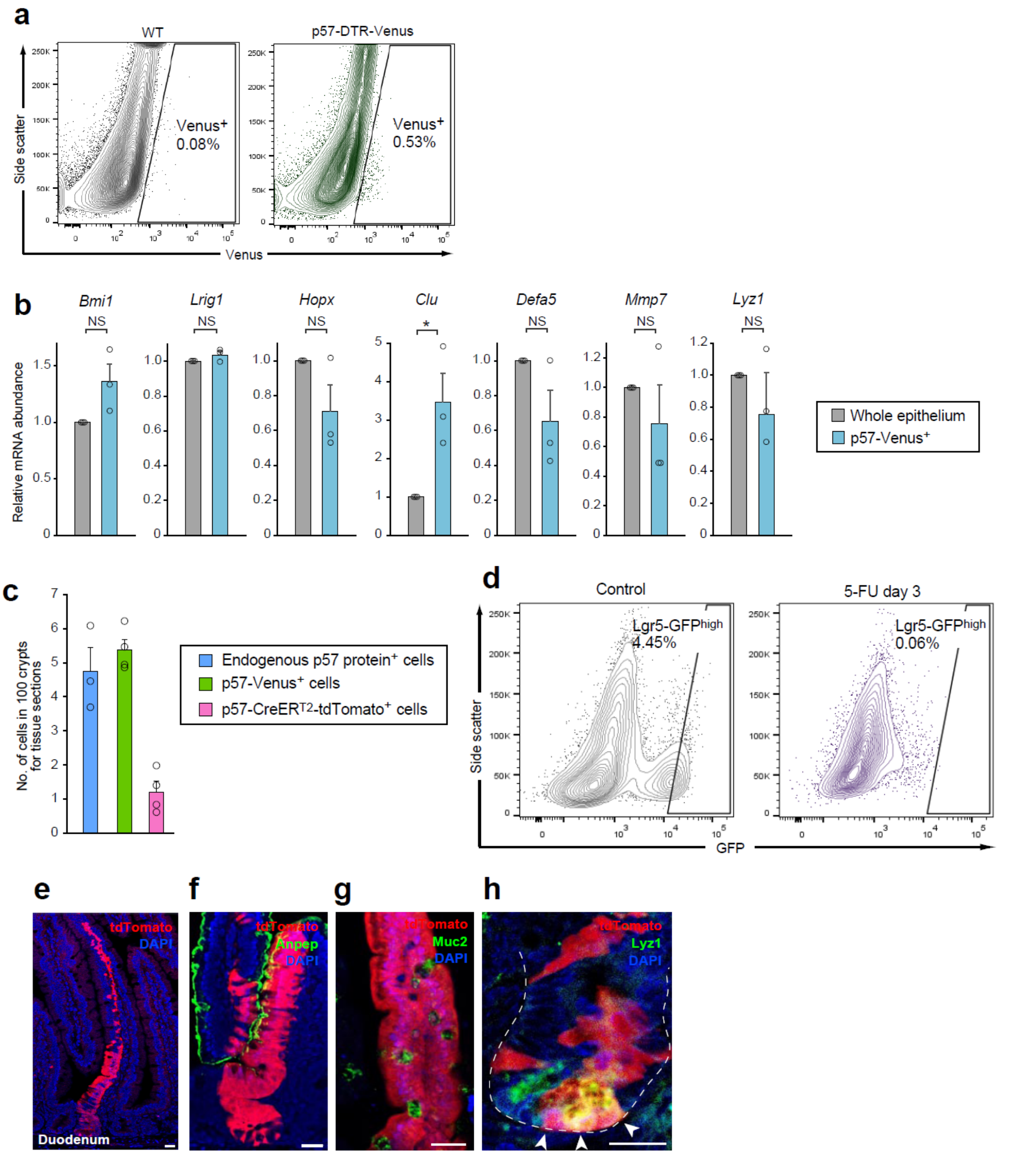

Supplementary Fig. 2 Characterization of p57-DTR-Venus and p57-CreER^{T2}/CAG-LSL-tdTomato mice as well as lineage tracing for intestinal p57^{+} cells. a Representative FACS plot for isolation of Venus^{+} crypt cells from intestinal epithelial cells of p57-DTR-Venus mice (right). A corresponding plot for WT mice is shown as a negative control (left). **b** RT-qPCR analysis of relative mRNA abundance for +4 ISC (*Bmi1, Lrig1*, and *Hopx*), revSC (*Clu*), or Paneth cell (*Defa5*, *Mmp7* and *Lyz1*) markers in sorted p57-Venus^{+} or all crypt cells (n = 3 mice). **c** Quantitative analysis of tissue sections for the number of cells stained with antibodies to p57 in WT mice (endogenous p57 protein^{+} cells) (n =3 mice), of Venus^{+} cells in p57-DTR-Venus mice (p57-Venus^{+} cells) (n =4 mice), and of tdTomato^{+} cells in p57-CreER^{T2}/CAG-LSL-tdTomato mice at 24 h after injection of tamoxifen at 50 mg/kg (p57-CreER^{T2}-tdTomato^{+} cells) (n =4 mice). **d** Representative FACS plots for the proportion of Lgr5-GFP^{high} cells among intestinal epithelial cells of Lgr5-EGFP-IRES-CreER^{T2} mice 3 days after injection of vehicle (left) or 5-FU at 150 mg/kg (right). **e–h** Representative immunofluorescence images of 5-FU–induced p57^{+} cell–derived clones in the duodenum (**e**) or of such clones containing Anpep^{+} enterocytes (**f**), Muc2^{+} goblet cells (**g**), or Lyz1^{+} Paneth cells (**h**). The dashed line shows the crypt outline. Arrowheads indicate tdTomato-labeled Lyz1^{+} Paneth cells. Scale bars, 20 µm. Quantitative data in **b** and **c** are means + SEM. NS (not significant), *P < 0.05 (two-tailed Student's t test). Source data are provided as a Source Data file.

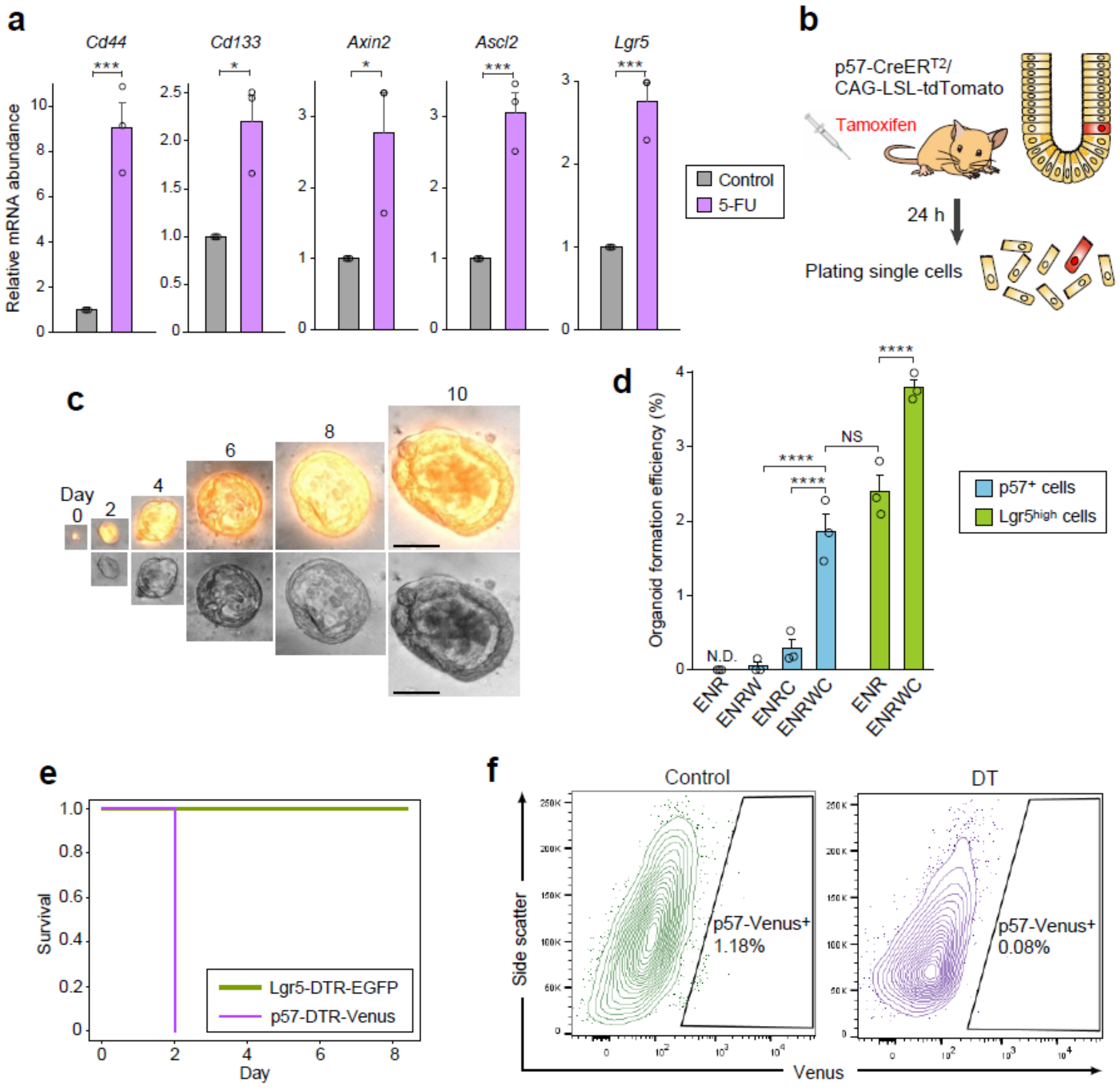

Supplementary Fig. 3 Wnt-induced acquisition of stemness in p57$^+$ crypt cells in vitro. a RT-qPCR analysis of relative mRNA abundance for Wnt signaling–related genes in whole-crypt cells derived from WT mice 3 days after injection of vehicle or 5-FU at 150 mg/kg (n = 3 mice). *$P < 0.05$, ***$P < 0.005$ (two-tailed Student's t test). **b** Schematic representation of the experimental protocol for evaluation of organoid formation from single p57$^+$ cells in vitro. **c** Representative 10-day tracing images (tdTomato fluorescence and differential interference contrast) of a growing organoid derived from a single p57$^+$ cell and labeled entirely with tdTomato in ENRWC medium. Scale bars, 20 μm. **d** Efficiency of organoid formation from single p57$^+$ cells under the indicated culture conditions at 5 days after plating (n = 3 independent experiments). Lgr5^{high} cells similarly isolated from tamoxifen-treated Lgr5-EGFP-IRES-CreER^{T2}/CAG-LSL-tdTomato mice were also examined. NS (not significant), ****$P < 0.001$ (Tukey-Kramer test). N.D., not detected. **e** Survival curves for Lgr5-DTR-EGFP (n = 4) or p57-DTR-Venus (n = 4) mice injected intraperitoneally with 50 μg/kg DT at day 0. **f** Representative FACS plots for cells from p57-DTR-Venus organoids cultured in the presence of vehicle (left) or 5 ng/ml DT (right) for 2 days. Depletion of p57-Venus$^+$ cells is apparent in the sample from DT-treated organoids. Data in **a** and **d** are means + SEM. Source data are provided as a Source Data file.

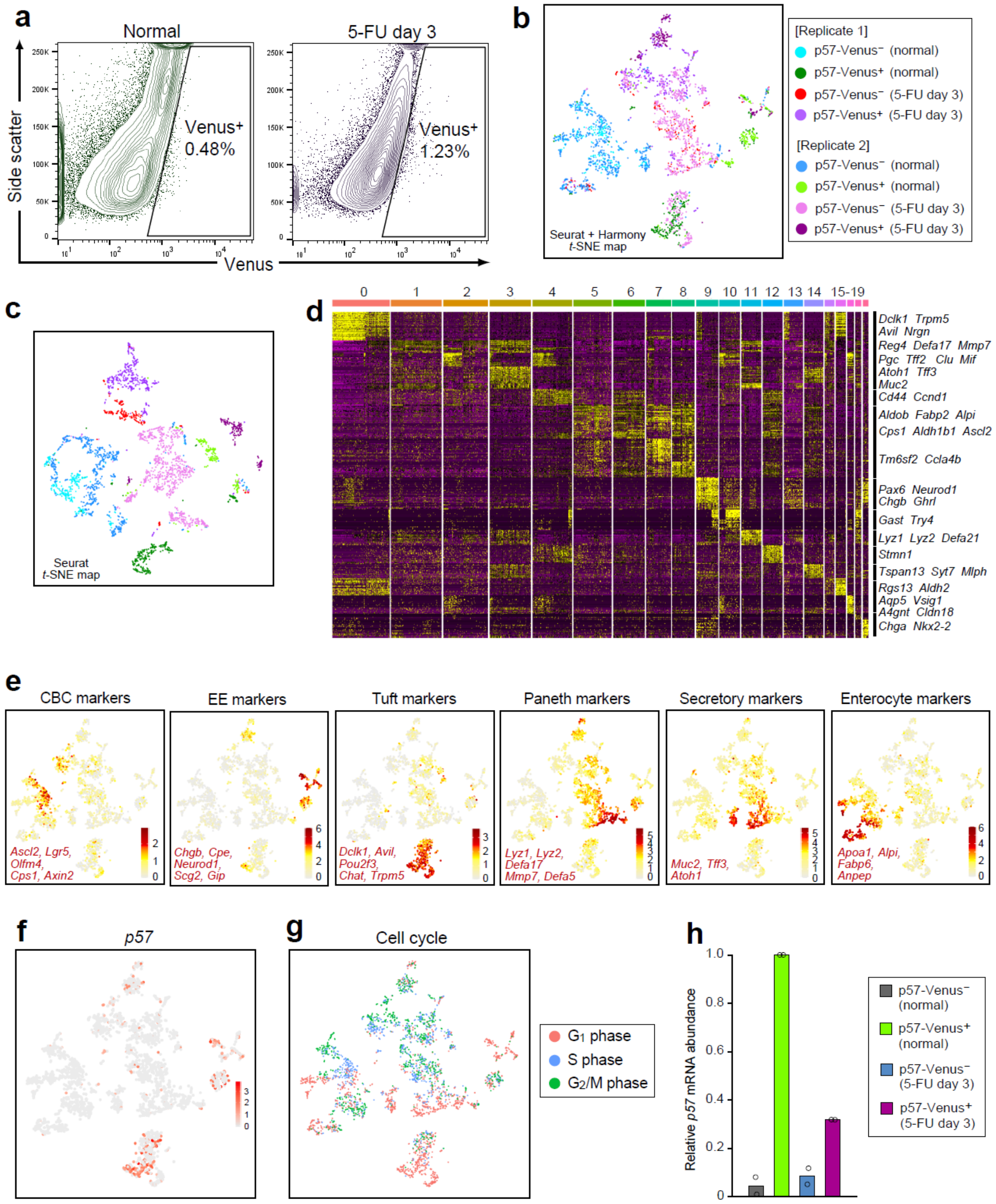

Supplementary Fig. 4 scRNA-seq analysis of normal and postinjury crypt cells from p57-DTR-Venus mice. a Representative FACS plots for crypt cells isolated from p57-DTR-Venus mice at 3 days after injection with vehicle (left) or 5-FU at a dose of 150 mg/kg (right). **b**, **c** *t*-SNE maps of single crypt cells from normal and postinjury p57-DTR-Venus mice as computed by Seurat in combination with Harmony software (**b**) or by simple clustering analysis in Seurat (**c**). Cells are color coded on the basis of p57-Venus expression status, experimental condition, and the biological replicate from which they were derived. **d** Heat map showing the z-score for expression level of each of the cluster-specific markers identified by Seurat. The known cell type–specific markers identified therein are indicated. **e** Heat maps for the mean expression level of the indicated cell type–specific genes—CBC, EE cell, tuft cell, Paneth cell, secretory cell, or enterocyte markers—shown on the *t*-SNE projection. **f** Heat map for the abundance of p57 mRNA shown on the *t*-SNE

projection. **g** Seurat-based cell cycle analysis of the crypt cell scRNA-seq data overlaid on the *t*-SNE map. **h** RT-qPCR analysis of relative p57 mRNA abundance in the p57-Venus^{-} and p57-Venus^{+} crypt cells from normal or postinjury mice subjected to the scRNA-seq analysis in Figure 4. Data are means (n = 2 biological replicates). Source data are provided as a Source Data file.

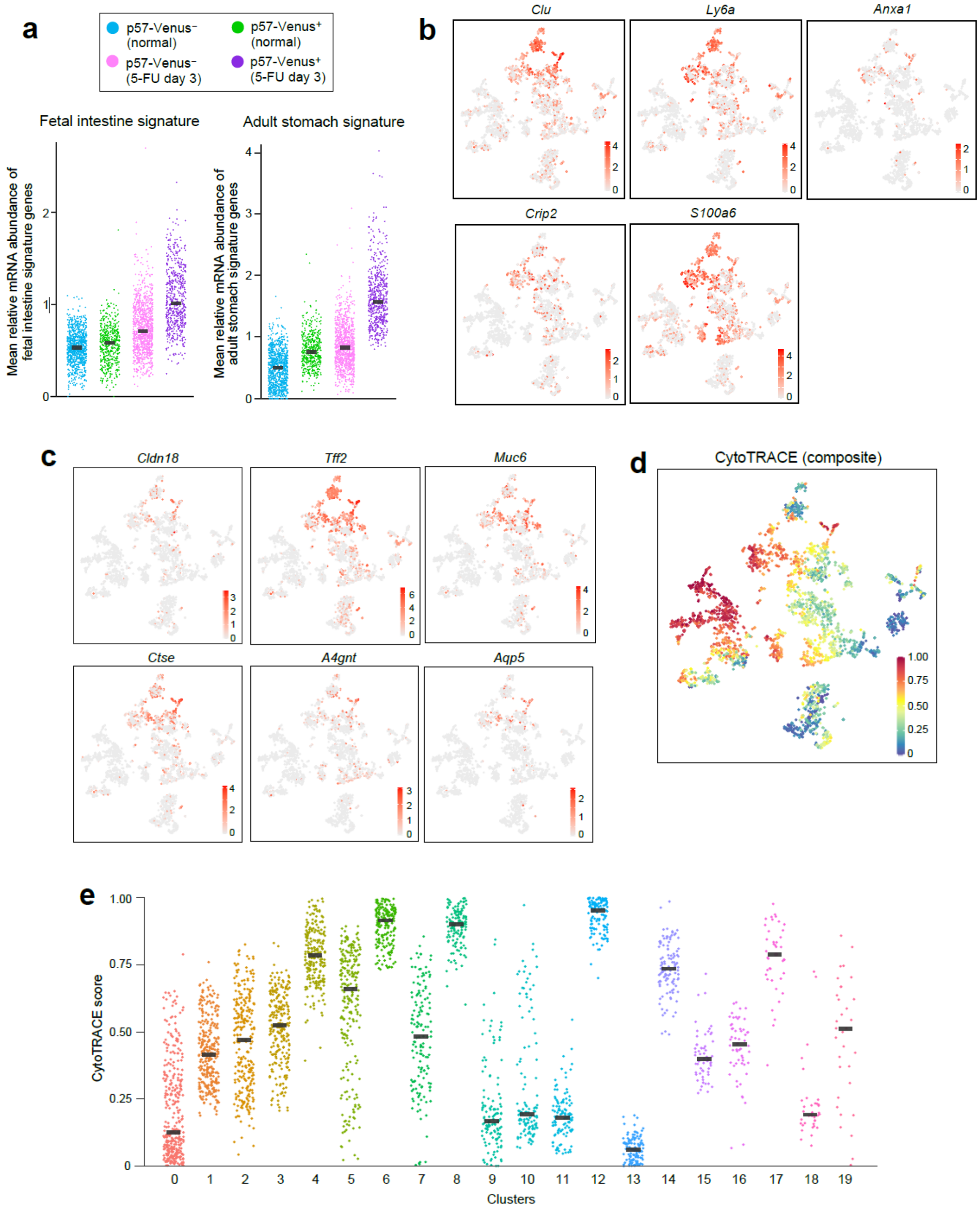

Supplementary Fig. 5 Expression of fetal intestine– or adult stomach–specific genes and CytoTRACE analysis for the crypt scRNA-seq data. a Relative mean mRNA abundance for fetal intestine (left) or adult stomach (right) signature genes in scRNA-seq analysis of normal and postinjury crypt cells. Cells are grouped and color coded on the basis of p57-Venus expression status and experimental condition. **b**, **c** Heat maps for the expression level of fetal intestine–specific genes (**b**) or adult stomach–specific genes (**c**) shown on the *t*-SNE map. **d** A composite heat map for the CytoTRACE scores of normal and postinjury crypt cells shown in Figure 5a is overlaid on the *t*-SNE projection. **e** CytoTRACE scores of normal and postinjury crypt cells. Cells are grouped and color coded on the basis of the unsupervised clusters identified by Seurat/Harmony. Source data are provided as a Source Data file.

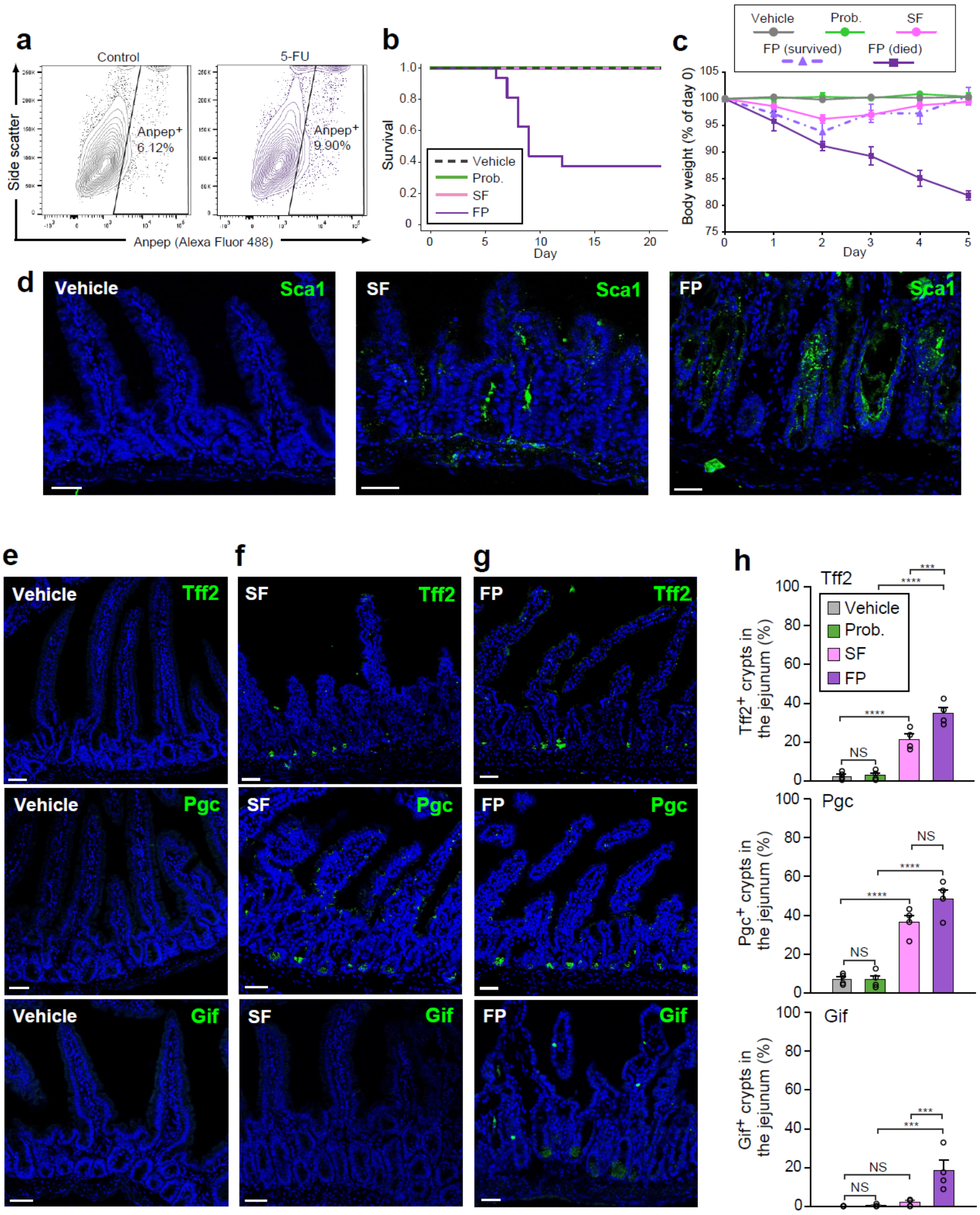

Supplementary Fig. 6 Characterization of spatiotemporal reprogramming at the population and histological levels. a Representative FACS plots for Anpep staining of crypt cells isolated from WT mice at 3 days after injection with vehicle (left) or 5-FU at a dose of 150 mg/kg (right). **b** Survival curves for WT mice treated with vehicle (*n* = 4), probenecid (Prob., *n* = 6), the SF protocol (*n* = 8), or the FP protocol (*n* = 16). **c** Time course of body weight for WT mice treated with vehicle (*n* = 3), probenecid (*n* = 4), or the SF protocol (*n* = 4) or for those that survived (*n* = 4) or died (*n* = 5) after treatment with the FP protocol. Data are means ± SEM. **d** Representative immunofluorescence staining of Sca1 in the ileum of WT mice treated with vehicle, the SF protocol, or the FP protocol. **e**–**g** Representative immunofluorescence staining of Tff2, Pgc, or Gif in the jejunum of mice treated with vehicle (**e**), the SF protocol (**f**), or the FP protocol (**g**). **h** Quantification of the fraction of $Tff2^+$, Pgc^+, or Gif^+ crypts in the jejunum of mice treated with vehicle, with probenecid alone (Prob., images not shown), with the SF protocol, or with the FP protocol determined from images as in **e** to **g**. Data are means + SEM. Four mice and 200 crypts per mouse were analyzed for each condition. NS (not significant), ****P* < 0.005, *****P* < 0.001 (Tukey-Kramer test). Scale bars, 50 µm. Nuclei were stained with DAPI (blue) in immunofluorescence images. Source data are provided as a Source Data file.

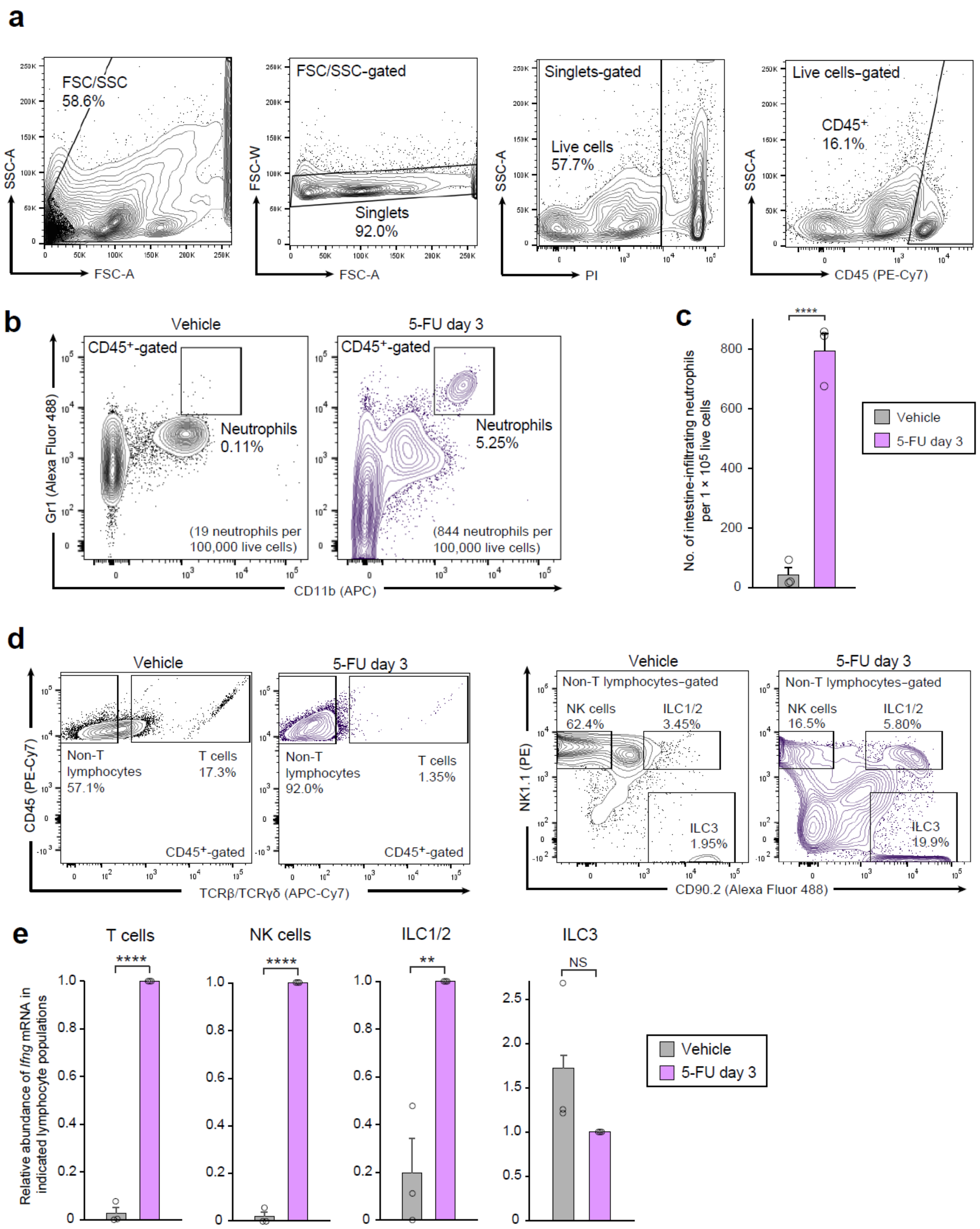

Supplementary Fig. 7 Activation of interferon-γ signaling in intestinal immune cells after injury with 5-FU. a Representative FACS plots showing gating strategy for the CD45^{+} hemocyte fraction in mouse intestine. FSC, forward scatter; SSC, side scatter. **b** Representative FACS plots showing the Gr1^{+}CD11b^{+} neutrophil fraction in the intestine of WT mice at day 3 after treatment with vehicle or 5-FU at 150 mg/kg. **c** The number of neutrophils per 1×10^5 total live cells in the intestine calculated from plots as in **b** ($n = 3$ mice). **d** Representative FACS gating strategy for sorting of TCR^{+} T cells, NK1.1^{+}CD90.2^{-} NK cells, as well as NK1.1^{+}CD90.2^{+} ILC1/2 and NK1.1^{-}CD90.2^{+} ILC3 fractions in the mouse intestine. **e** RT-qPCR analysis of relative *Ifng* mRNA abundance in the indicated lymphocyte fractions sorted from WT mice 3 days after treatment with vehicle or 5-FU at 150 mg/kg ($n = 3$ mice). Data in **c** and **e** are means + SEM. NS (not significant), $^{**}P < 0.01$, $^{****}P < 0.001$ (two-tailed Student's t test). Source data are provided as a Source Data file.

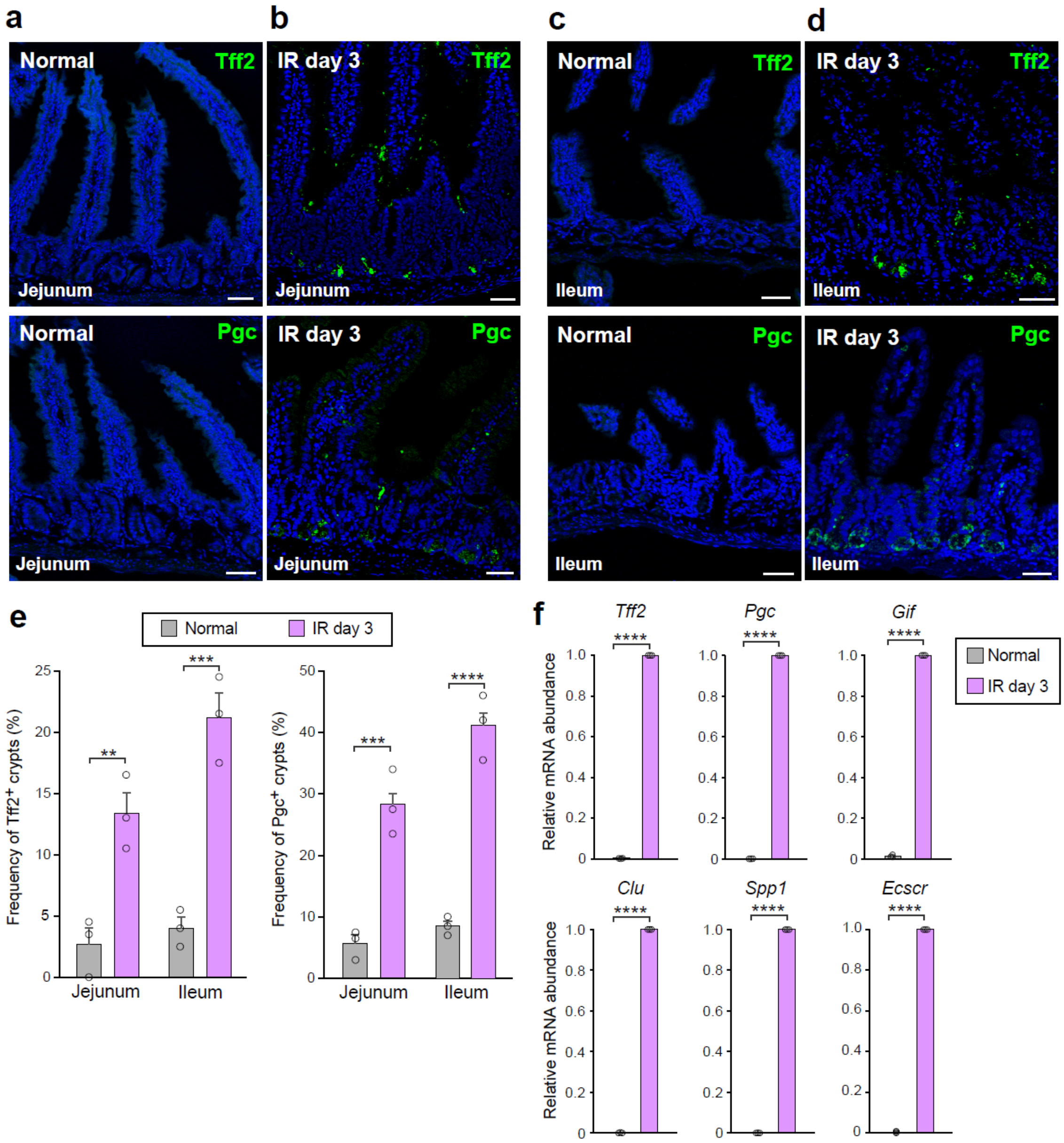

Supplementary Fig. 8 Metaplasia-like changes of the intestinal epithelium during regeneration from IR-induced damage. a–**d** Representative immunofluorescence images of Tff2 or Pgc staining in the jejunum of normal WT mice (**a**) or WT mice at 3 days after exposure to 8.5 Gy of IR (**b**) as well as in the ileum of normal WT mice (**c**) or WT mice 3 days after exposure to 8.5 Gy of IR (**d**). Nuclei were stained with DAPI (blue). Scale bars, 50 µm. **e** Quantification of the fraction of Tff2$^+$ (left) or Pgc$^+$ (right) crypts in the jejunum or ileum of control mice or those at 3 days after exposure to 8.5 Gy of IR determined from images as in **a** to **d**. Microscopic fields were randomly selected, and crypts containing at least three marker-positive cells were regarded as marker-positive crypts (n = 3 mice and 200 crypts per mouse were analyzed for each condition). **f** RT-qPCR analysis of relative mRNA abundance for gastric markers in intestinal crypt cells from control mice or those at 3 days after exposure to 12 Gy of IR (n = 3 mice). Quantitative data are means + SEM. NS (not significant), $^{**}P < 0.01$, $^{***}P < 0.005$, $^{****}P < 0.001$ (two-tailed Student's t test). Source data are provided as a Source Data file.

Supplementary Table Information

Supplementary Table 1

This file contains information on gene sets used for analysis of scRNA-seq data.

Supplementary Table 2

This file contains information on primer sequences used in RT-qPCR analysis.

Supplementary Table 3

This file contains information on sequences for CEL-seq2 RT primers containing UMIs and cell barcodes.
